# Distinct clinical phenotypes in paediatric cancer patients with sepsis are associated with different outcomes—an international multicentre retrospective study

**DOI:** 10.1016/j.eclinm.2023.102252

**Published:** 2023-10-05

**Authors:** Roelie M. Wösten-van Asperen, Hannah M. la Roi-Teeuw, Rombout BE. van Amstel, Lieuwe DJ. Bos, Wim JE. Tissing, Iolanda Jordan, Christian Dohna-Schwake, Gabriella Bottari, John Pappachan, Roman Crazzolara, Rosanna I. Comoretto, Agniezka Mizia-Malarz, Andrea Moscatelli, María Sánchez-Martín, Jef Willems, Colin M. Rogerson, Tellen D. Bennett, Yuan Luo, Mihir R. Atreya, E.Vincent S. Faustino, Alon Geva, Scott L. Weiss, Luregn J. Schlapbach, L Nelson Sanchez-Pinto, POKER (PICU Oncology Kids in Europe Research group) research consortium of ESPNIC (European Society of Paediatric & Neonatal Intensive Care), and, Marina Caballero, Adriana Margarit, Roi Campos, Paula Möller, Carmela Serpe, Angela Amigoni, Maria Damps, Alessia Montaguti, Giacomo Tardini, the Novel Data-Driven Sepsis Phenotypes in Children study groupab, Juliane Bubeck-Wardenburg, Reid Farris Farris, Mark Hall, Grace Chong, Sareen Shah, Robinder Khemani, Emily Stroup

**Affiliations:** a Department of Paediatric Intensive Care, University Medical Centre Utrecht/Wilhelmina Children’s Hospital, Utrecht, the Netherlands; b Intensive Care, Amsterdam UMC—location AMC, University of Amsterdam, Amsterdam, the Netherlands; c Princess Máxima Centre for Pediatric Oncology, Utrecht, the Netherlands; d Department of Paediatric Oncology, University of Groningen, University Medical Centre Groningen, Groningen, the Netherlands; e Department of Paediatric Intensive Care and Institut de Recerca, Hospital Sant Joan de Déu, University of Barcelona, Barcelona, Spain; f Consorcio de Investigación Biomédica en Red de Epidemiología y Salud Pública, Madrid, Spain; g Department of Paediatrics I, Paediatric Intensive Care, Children’s Hospital Essen, Germany; h West German Centre for Infectious Diseases, University Hospital Essen, University Duisburg-Essen, Essen, Germany; i Paediatric Intensive Care Unit, Children’s Hospital Bambino Gesù, IRCSS, Rome, Italy; j Department of Paediatric Intensive Care, Southampton Children’s Hospital, UK; k Department of Paediatrics, Paediatric Intensive Care Unit, Medical University of Innsbruck, Innsbruck, Austria; l Department of Paediatric Intensive Care, Department of Woman's and Child's Health, Padua University Hospital, Padua, Italy; m Department of Paediatric Oncology, Haematology and Chemotherapy Unit, Medical University of Silesia, Katowice, Poland; n Neonatal and Paediatric Intensive Care Unit, IRCCS Istituto Giannina Gaslini, Genova, Italy; o Department of Paediatric Intensive Care, Hospital Universitario La Paz, Madrid, Spain; p Department of Paediatric Intensive Care, Ghent University Hospital, Ghent, Belgium; q Department of Paediatrics, Division of Critical Care, Indianapolis University School of Medicine, Indianapolis, IN, USA; r Departments of Biomedical Informatics and Paediatrics, University of Colorado School of Medicine, Aurora, CO, USA; s Department of Preventive Medicine, Northwestern University Feinberg School of Medicine, Chicago, IL, USA; t Department of Paediatrics (Critical Care), University of Cincinnati College of Medicine, Cincinnati Children’s Hospital Medical Centre, Cincinnati, OH, USA; u Department of Paediatrics, Yale School of Medicine, New Haven, CT, USA; v Department of Anaesthesiology, Critical Care, and Pain Medicine and Computational Health Informatics Program, Boston Children's Hospital, USA; w Department of Anaesthesia, Harvard Medical School, Boston, MA, USA; x Division of Critical Care, Department of Paediatrics, Nemours Children’s Health, Delaware, USA; y Department of Intensive Care and Neonatology and Children’s Research Centre, University Children’s Hospital Zurich, University of Zurich, Zurich, Switzerland; z Child Health Research Centre, The University of Queensland, Brisbane, Queensland, Australia; aa Department of Paediatrics (Critical Care) and Preventive Medicine (Health & Biomedical Informatics), Northwestern University Feinberg School of Medicine and Ann & Robert H Lurie Children’s Hospital of Chicago, Chicago, IL, USA

**Keywords:** Paediatric intensive care, Oncology, Sepsis, Phenotype, Latent class analysis

## Abstract

**Background:**

Identifying phenotypes in sepsis patients may enable precision medicine approaches. However, the generalisability of these phenotypes to specific patient populations is unclear. Given that paediatric cancer patients with sepsis have different host response and pathogen profiles and higher mortality rates when compared to non-cancer patients, we determined whether unique, reproducible, and clinically-relevant sepsis phenotypes exist in this specific patient population.

**Methods:**

We studied patients with underlying malignancies admitted with sepsis to one of 25 paediatric intensive care units (PICUs) participating in two large, multi-centre, observational cohorts from the European SCOTER study (n = 383 patients; study period between January 1, 2018 and January 1, 2020) and the U.S. Novel Data-Driven Sepsis Phenotypes in Children study (n = 1898 patients; study period between January 1, 2012 and January 1, 2018). We independently used latent class analysis (LCA) in both cohorts to identify phenotypes using demographic, clinical, and laboratory data from the first 24 h of PICU admission. We then tested the association of the phenotypes with clinical outcomes in both cohorts.

**Findings:**

LCA identified two distinct phenotypes that were comparable across both cohorts. Phenotype 1 was characterised by lower serum bicarbonate and albumin, markedly increased lactate and hepatic, renal, and coagulation abnormalities when compared to phenotype 2. Patients with phenotype 1 had a higher 90-day mortality (European cohort 29.2% versus 13.4%, U.S. cohort 27.3% versus 11.4%, p < 0.001) and received more vasopressor and renal replacement therapy than patients with phenotype 2. After adjusting for severity of organ dysfunction, haematological cancer, prior stem cell transplantation and age, phenotype 1 was associated with an adjusted OR of death at 90-day of 1.9 (1.04–3.34) in the European cohort and 1.6 (1.2–2.2) in the U.S. cohort.

**Interpretation:**

We identified two clinically-relevant sepsis phenotypes in paediatric cancer patients that are reproducible across two international, multicentre cohorts with prognostic implications. These results may guide further research regarding therapeutic approaches for these specific phenotypes.

**Funding:**

Part of this study is funded by the 10.13039/100009633Eunice Kennedy Shriver National Institute of Child Health and Human Development.

**Research in context**

**Evidence before this study**

Previous studies of patients with sepsis, both in adults and children, have identified phenotypes with differential patient outcomes and responses to treatment. Although patients with underlying malignancies suffer from the highest sepsis-related mortality amongst critically ill children, they are often under-represented or excluded in previous phenotyping studies. Whether specific phenotypes can also be identified in paediatric cancer patients with sepsis is unknown. A PubMed search using the terms sepsis AND (cancer OR oncology) AND (subtype OR subphenotype OR endotype OR phenotype) without language restrictions identified no previous studies.

**Added value of this study**

In the present study, in which we analysed two independent cohorts of a total of 2281 children with malignancies presenting to a PICU with sepsis, we identified two phenotypes. Phenotype 1 was characterised by more severe organ dysfunction pattern when compared to phenotype 2. Assignment to phenotype 1 was associated with worse clinical outcomes, including higher PICU resource use and mortality, and that association persisted after adjusting for common confounders, including the severity of organ dysfunction, suggesting that this approach is not simply stratifying patients by severity on presentation.

**Implications of all the available evidence**

These findings provide proof-of-concept that the population of paediatric cancer patients with sepsis contains distinct phenotypes with significantly different outcomes. Future studies should aim to further elucidate the pathobiological pathways underpinning these phenotypes and identifying potential therapeutic targets, such as immunomodulatory drugs, that could result in more personalised care in this high-risk population.

## Introduction

Oncology has become one of the first disciplines to deliver highly personalised treatment to many patients, enabling breakthrough improvements in cancer-related survival.^1^^,^^2^ The estimated burden of paediatric cancer nowadays is 413 000 new cases of children with cancer worldwide, with an expected growth of 13.7 million cases of childhood cancer between 2020 and 2050.^3^ Currently, 5-year overall survival in high-income countries has increased to almost 80% in children and adolescents.^4^^,^^5^ Accordingly, there has been a shift from primarily cancer-related to treatment-related deaths. Today, infections represent the leading causes of non-cancer-related mortality and morbidity in oncologic children.^6^^,^^7^ In particular, the progression towards organ dysfunction and requirement for treatment in the intensive care unit (ICU) remains associated with high mortality rates, ranging from 20 to 30%, substantially higher than sepsis mortality in non-cancer critically ill children.^8^, ^9^, ^10^, ^11^, ^12^

Sepsis is a heterogeneous syndrome. Recently, phenotypes have been identified in both adult and paediatric sepsis patients using data-driven approaches.^13^, ^14^, ^15^ Four phenotypes were identified characterized by different demographics, laboratory values, and patterns of organ dysfunction.^13^^,^^14^ The phenotypes identified in paediatric sepsis patients resemble the characteristics of the phenotypes in adult sepsis patients. In both studies, the phenotypes were all strongly correlated with distinctive patterns of the host immune response with substantial differences in inflammatory mediators (IL-6, IL-10, IL-8, and TNF-α) and coagulation between the different phenotypes.^13^^,^^14^ In addition, they were associated with different outcomes and differential responses to therapy.

Paediatric cancer patients with sepsis are very different in terms of host response and responsible pathogens^16^ and have much higher mortality rates than septic children without cancer.^16^, ^17^, ^18^ These patients also demonstrated significant heterogeneity and there may be phenotypes that are unique to this specific patient population. Stratification into different phenotypes may facilitate the discovery of specific biological pathways that may be susceptible to targeted therapies, which is a crucial step towards personalised medicine.

In this study, we aimed to determine the existence of different phenotypes in children with cancer admitted to the PICU with sepsis using latent class analysis of two international multicentre cohorts. We hypothesised that distinct phenotypes exist in paediatric cancer patients with sepsis and that these phenotypes are reproducible and have prognostic relevance.

## Methods

### Observational cohorts

To test the hypothesis that different phenotypes exist among paediatric cancer patients admitted to PICU with sepsis, we performed latent class analysis in two retrospective observational cohorts. The first was the European *Subphenotyping Children with Oncological diseases TrEated at the PICU for infections and inflammatory conditions: a Retrospective* (SCOTER) study, a retrospective multicentre study of paediatric cancer patients with sepsis admitted to the PICUs of 12 participating hospitals from the POKER consortium (PICU Oncology Kids in Europe research group), a working group of the European Society for Paediatric and Neonatal Intensive Care (ESPNIC), between January 1, 2018 and January 1, 2020. All consecutive patients admitted to the PICU with age <18 years, a diagnosis of malignancy according to ICD-10 code, a suspected infection and SIRS according to the 2005 International Pediatric Sepsis Consensus Conference criteria were included.^19^ Patients with treatment limitation orders or lack of consent for research-related use of patient’s health data were excluded. In this study, data were collected on demographics, medical history, clinical parameters, laboratory values, microbiology outcomes, and treatment within the first 24 h of PICU admission. The primary outcome was 90-day mortality. Secondary outcomes were PICU resource use, including mechanical ventilation, vasopressor use, continuous renal replacement therapy (CRRT) and extracorporeal membrane oxygenation (ECMO).

For the second cohort, we obtained data from the U.S. *Novel Data-Driven Sepsis Phenotypes in Children* study. This study was a retrospective, multicentre, observational cohort study of children 0–18 years old admitted to one of 13 participating PICUs in the U.S. between January 1, 2012 and January 1, 2018. Patients who had a confirmed or suspected infection (i.e., received systemic antimicrobials and microbiological testing in the ±24 h time-window after the first admission to the PICU) were included. For the purpose of the current study, data were extracted only from children with an underlying malignancy who met criteria for sepsis based on the combination of a confirmed or suspected infection and organ dysfunction on the day of admission (i.e., a paediatric Sequential Organ Failure Assessment [pSOFA] subscore of >1 in 2 or more organs). Analyses in both cohorts were done locally, such that no protected health information was exchanged between study sites.

This study followed the Strengthening the Reporting of Observational Studies in Epidemiology (STROBE) reporting guideline for cohort studies (Supplementary Appendix).

### Statistical analysis

We used latent class analysis (LCA) to identify phenotypes within each international cohort independently according to the approach described by Sinha and colleagues.^20^ LCA is a well validated statistical technique of finite mixture modelling that allows for identification of unobserved (latent) subgroups or classes that have a given probability of occurrence and are characterized by a specific and predictable combination of clinical variables and other features. LCA has the advantage that it defines these subgroups by considering multiple variables concurrently, independent of the outcomes.

Baseline demographic, clinical, and laboratory data were selected as potential class-defining variables in the LCA model based on their association with sepsis onset or outcome, as well as variables used in previous studies,^13^^,^^14^ and their availability in the electronic health records. In addition, we included as inputs in the LCA model characteristics of underlying malignancies (haematologic versus non-haematologic) and prior haematopoietic stem cell transplantation (HSCT), previously associated with poor clinical outcomes in paediatric cancer patients.^11^^,^^12^ Similar to previous studies,^13^^,^^14^^,^^21^ we used the worst values during the first 24 h of PICU admission for the clinical and laboratory variables. Some variables (including heart rate, respiratory rate, systolic blood pressure and creatinine) are age-dependent and we transformed these variables to z-scores based on age-categories as defined by the PODIUM criteria.^22^ We described details on variable selection, multiple imputation approach to missing data, standardisation of age-dependent variables, and a complete list of the variables included in the LCA model in the Supplementary Appendix.

We determined the optimal number of classes (k) using a combination of criteria, including entropy, Bayesian Information and Akaike’s Information Criteria, and adequate sample size within each class. Five models, comprising 1 to 5 classes, were fitted. Once we established the number of classes, we assigned the patients to their most likely class. Subsequently, we performed an LCA in the U.S. cohort and compared the characteristics of the resulting phenotypes with the European cohort.

We evaluated the prognostic value of the phenotypes by comparing differences in 90-day mortality, requirement of ventilator- and vasopressor support, continuous renal replacement therapy (CRRT) and extracorporeal membrane oxygenation (ECMO) between the phenotypes and across the two international cohorts. Additionally, we determined whether class membership was independently associated with 90-day mortality by using logistic regression after adjusting for known confounders of outcomes, including type of malignancy (haematologic versus non-haematologic), prior HSCT, age, and organ dysfunction burden on admission (based of the pSOFA score).

To assess the robustness of the reproducibility of the phenotypes across the two international cohorts, we performed a sensitivity analysis by training a classifier on the European cohort and assessing the overlap of the predicted phenotypes with the LCA results in the U.S. cohort. Briefly, a Random Forest classifier was trained in the European cohort with the outcome being the phenotype and the features being the same class-defining variables used in the LCA. We then used the resulting Random Forest model to assign a predicted phenotype in the U.S. cohort and assessed the inter-rater agreement between the predicted phenotype and the LCA-based phenotype.

Patient data are presented as mean and SD for normally distributed continuous variables, median and IQR for variables that were not normally distributed, and numbers with percentages for categorical data. Differences between identified phenotypes were tested using t-test, Mann–Whitney U test, and Fisher’s exact test, as appropriate. We used the Kaplan–Meier method with the use of the log-rank test to assess the unadjusted association between phenotype classification and 90-day mortality. We used R version 4.2.1. for the statistical analyses.

### Ethics statement

The study activities were approved by the institutional review boards at all participating sites. We obtained written informed consent for the use of clinical data or a waiver from informed consent was granted by the ethics commission, depending on the requirements of the participating sites.

### Role of the funding source

The funder of the study had no role in study design, data collection, data analysis, data interpretation, or writing of the report. RMWvA, HMlRT, and LNSP had access to the data. All authors decided to publish the study findings.

## Results

The European cohort included 383 patients and the U.S. cohort included 1898 patients. Baseline clinical characteristics for patients in both cohorts were generally comparable, with some exceptions (Supplementary Table S2). In both cohorts, haematological malignancies were the predominant underlying malignancy, but the European cohort comprised more haematological malignancies patients (73%) than the U.S. cohort (61%). In addition the European cohort included more HSCT recipients (29%) when compared to the U.S. cohort (17%).

LCA demonstrated that a two-class model provided the best fit for the European cohort (Supplementary Fig. S1 and Table S3). Entropy in all models was 0.75 or greater, indicating adequate class separation. Average latent class probabilities were 0.97 for phenotype 1 (87.6% with probability >90%) and 0.97 for phenotype 2 (90.7% with probability >90%) (Supplementary Fig. S2). The values of both the Bayesian Information and Akaike’s Information Criteria continued to decrease as the number of classes increased, which may indicate that the addition of more classes would improve the model fit. However, both indices showed a point of inflection at two classes. These findings led us to proceed using a two-class model to characterise two phenotypes.

**Fig. 1:**
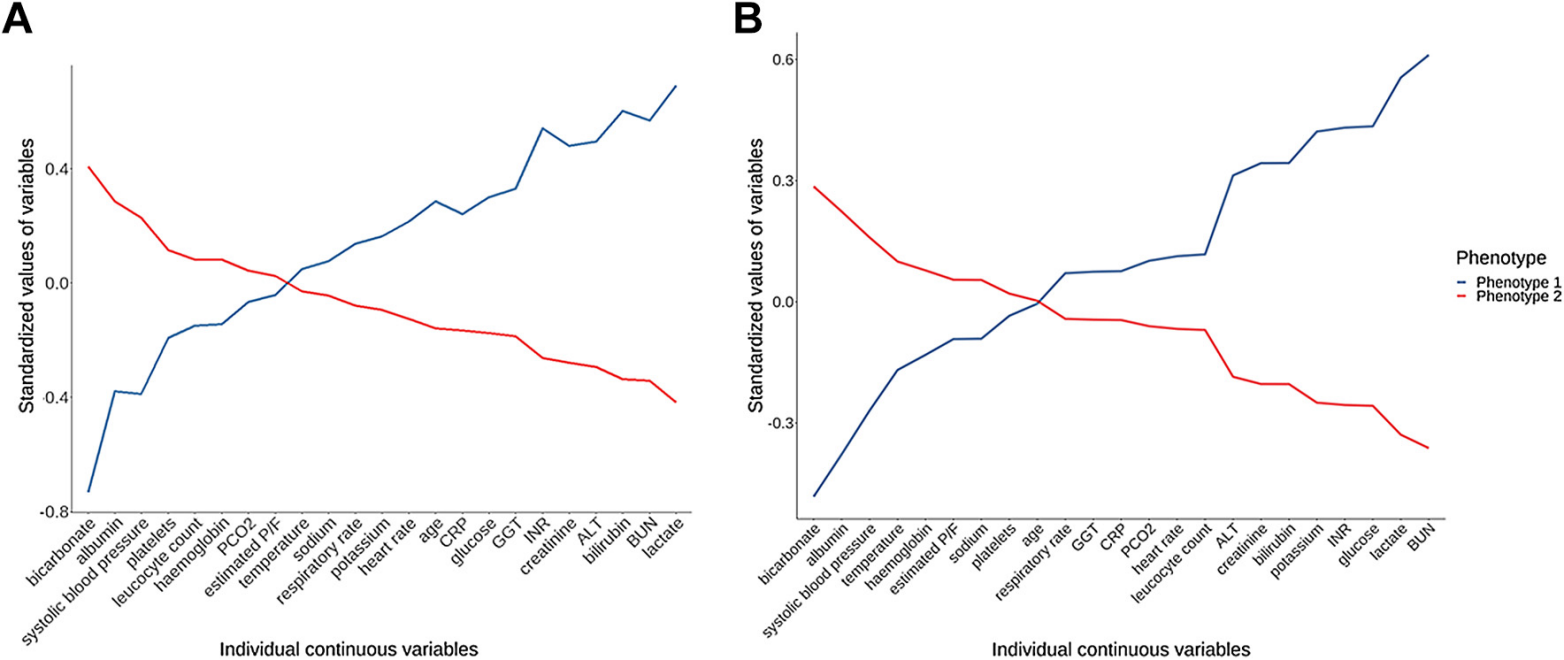
**Differences in standardised values of the continuous variables by phenotype in the European cohort (A) and the U.S. cohort (B).** The variables are sorted on the basis of the degree of separation between the phenotypes from maximum positive separation on the left (i.e., phenotype 2 higher than phenotype 1) to maximum negative separation on the right (i.e., phenotype 2 lower than phenotype 1). The y-axis represents standardised variable values, in which all means are scaled to 0 and SDs to 1. A value of +1 for the standardised variable signifies that the mean value for a given phenotype was one SD higher than the mean value in the cohort as a whole. ALT = Alanine transaminase. BUN = Blood urea nitrogen. CRP =C-reactive protein. GGT = Gamma-glutamyltransferase.

To characterise the phenotypes, we assigned the patients to their most likely phenotype and examined the values of the variables used in the models for each phenotype (Table 1). In the European cohort, 137 (36%) patients were assigned to phenotype 1 and 246 (64%) to phenotype 2. Compared with phenotype 2, phenotype 1 was characterised by lower values of bicarbonate, albumin, markedly increased hepatic, renal, and coagulation abnormalities, increased levels of CRP, increased levels of lactate, low blood pressure and high heart rate (Fig. 1). In addition, patients with phenotype 1 showed haematological abnormalities with low haemoglobin concentration, low number of platelets and leucocytes when compared to phenotype 2. No differences between the phenotypes were found regarding underlying malignancies, prior HSCT, and gender.

**Table 1:** Characteristics of the phenotypes across the two cohorts.

Characteristic	European cohort			US cohort		
	Phenotype 1 N = 137 (35.8)	Phenotype 2 N = 246 (64.2)	p	Phenotype 1 N = 706 (37.2)	Phenotype 2 N = 1192 (62.8)	p
Demographics						
Age, months, median (IQR)	134 (75–182)	84 (36–154)	<0.001	99 (44–159)	94 (41–164)	0.92
Sex, male, No. (%)	89 (65.0)	128 (52.0)	0.02	396 (56.1)	667 (56.0)	0.99
Oncological diagnosis, No. (%)			0.31			<0.001
Haemato-oncological	109 (79.6)	172 (70.0)		473 (67.0)	681 (57.1)	
Solid tumour	17 (12.4)	31 (12.6)		128 (18.1)	228 (19.1)	
Brain & Central nervous system	9 (6.6)	42 (17.1)		87 (12.3)	245 (20.1)	
Other	2 (1.5)	1 (0.4)		14 (2.0)	27 (2.2)	
Prior HSCT, No. (%)	42 (30.7)	70 (28.5)	0.76	140 (19.8)	180 (15.1)	0.009
Vital parameters, median (IQR)						
Highest heart rate (bpm)	156 (135–170)	151 (130–171)	0.37	161 (142–179)	154 (136–175)	<0.001
Lowest systolic pressure (mmHg)	80 (69–48)	89 (77–101)	<0.001	76 (60–90)	83 (72–92)	<0.001
Highest respiratory rate (bpm)	40 (30–48)	38 (29–50)	0.9	42 (34–54)	40 (31–53)	0.003
Highest temperature (Deg. C)	38.0 (37.1–39.1)	38.0 (37.3–38.6)	0.54	38.5 (37.6–39.4)	39 (38–39.7)	<0.001
Laboratory parameters, median (IQR)						
Lowest estimated PaO_2_/FiO_2_	219 (143–437)	235 (138–448)	0.68	130 (91–216)	157 (94–235)	<0.001
Highest PCO_2_ (mmHg)	45 (38–51)	47 (42.0–51.0)	0.1	47 (40–59)	44 (38–52)	<0.001
Highest glucose (mmol/L)	8.9 (7.0–12.6)	7.5 (6.4–9.3)	<0.001	10.5 (7.5–15.7)	7.7 (6.3–10.2)	<0.001
Highest potassium (mmol/L)	4.3 (3.8–4.9)	4.1 (3.8–4.5)	0.03	4.2 (3.7–5.1)	3.9 (3.5–4.3)	<0.001
Highest sodium (mmol/L)	136 (132–140)	136 (134–139)	0.99	135 (131–138)	136 (133–138)	<0.001
Highest creatinine (μmol/L)	66.5 (37.3–120.3)	31.8 (22.1–49.0)	<0.001	70.7 (43.3–124)	34.5 (24.8–46.9)	<0.001
Highest BUN (mmol/L)	9.0 (6.1–13.7)	4.5 (3.0–6.6)	<0.001	8.2 (4.6–15)	3.6 (2.5–5.4)	<0.001
Highest albumin (g/L)	22 (18–29)	29 (24–34)	<0.001	25 (20–29)	28 (24–33)	<0.001
Lowest bicarbonate (mmol/L)	17.9 (14.6–21.3)	23.6 (21.3–26.0)	<0.001	18.0 (14.0–22.0)	22.0 (19.1–24.5)	<0.001
Highest lactate (mmol/L)	3.6 (1.7–5.6)	1.4 (1.0–2.1)	<0.001	3.8 (1.8–7.1)	1.4 (0.9–2.2)	<0.001
Highest bilirubin (μmol/L)	34.1 (16.9–82.7)	13.3 (6.8–22.1)	<0.001	30.8 (12.0–71.8)	13.7 (6.8–34.2)	<0.001
Highest ALT (U/L)	69 (30–181)	31.0 (18–58)	<0.001	84.5 (37.2–272)	35.0 (21.0–58.2)	<0.001
Highest GGT (U/L)	108.0 (52.0–372.3)	72.0 (25.8–146.3)	<0.001	76.5 (42–167)	74 (37–185)	0.63
Highest INR	1.5 (1.3–2.1)	1.2 (1.1–1.4)	<0.001	1.7 (1.4–2.2)	1.3 (1.1–1.5)	<0.001
Lowest WBC (10^9^/L)	1.06 (0.2–5.6)	2.1 (0.3–10.3)	0.06	3.5 (0.5–10.3)	2.8 (0.2–8.8)	0.003
Lowest platelets (10^9^/L)	19 (10–50)	28 (14–79)	0.01	43 (20–91)	52 (26–96)	0.001
Lowest haemoglobin (mmol/L)	4.8 (4.2–5.5)	5.1 (4.5–5.7)	0.02	5.2 (4.3–6.0)	5.4 (4.6–6.1)	<0.001
Highest CRP (mg/L)	195 (92–295)	134 (49–241)	0.001	105 (33–224)	76 (32–202)	0.247
Organ support						
Mechanical ventilation, No. (%)	74 (54.0)	111 (45.1)	0.11	435 (61.6)	569 (47.7)	<0.001
Vasopressor use, No. (%)	94 (68.6)	101 (41.1)	<0.001	461 (65.3)	558 (46.8)	<0.001
VIS score, median (IQR)	20 (14–53)	10 (7–20)	<0.001	20 (10–40)	10 (6–20)	<0.001
CRRT, No. (%)	30 (21.9)	7 (2.8)	<0.001	141 (20.0)	41 (3.4)	<0.001
ECMO, No. (%)	3 (2.2)	3 (1.2)	0.67	18 (2.5)	11 (0.9)	0.009
PELOD–2 score, median (IQR)	6 (4–9)	4 (2–6)	<0.001	8 (6–13)	6 (4–8)	<0.001
pSOFA score, median (IQR)	11 (9–13)	7 (5–10)	<0.001	9 (7–13)	7 (6–8)	<0.001
90–day mortality, No. (%)	40 (29.2)	33 (13.4)	<0.001	193 (27.3)	136 (11.4)	<0.001

ALT = Alanine transaminase; BUN = Blood urea nitrogen; CRP=C-reactive protein; CRRT = Continuous renal replacement therapy; ECMO = extracorporeal membrane oxygenation; GGT = Gamma-glutamyltransferase; HSCT = Haematopoietic Stem Cell Transplantation; IQR = interquartile range; PELOD = Paediatric Logistic Organ Dysfunction; pSOFA = Paediatric Sequential Organ Failure Assessment; VIS = Vaso-active inotropic score; WBC = White blood cell.

The LCA was repeated independently in the U.S. cohort. Also in the U.S. cohort, a two-class model provided the best fit (Supplementary Fig. S1 and Table S3). In this cohort, 706 (37%) and 1192 (63%) were assigned to phenotype 1 and 2, respectively. The characteristics of the two phenotypes in this cohort were generally similar to those in the European cohort, with phenotype 1 characterised by shock, high lactate, multiple organ dysfunctions, and comparable haematological abnormalities (Table 1). Additionally, patients assigned to phenotype 1 had lower estimated PaO_2_/FiO_2_-ratio’s compared to phenotype 2, whereas no difference was found between both phenotypes in the European cohort.

**Fig. 2:**
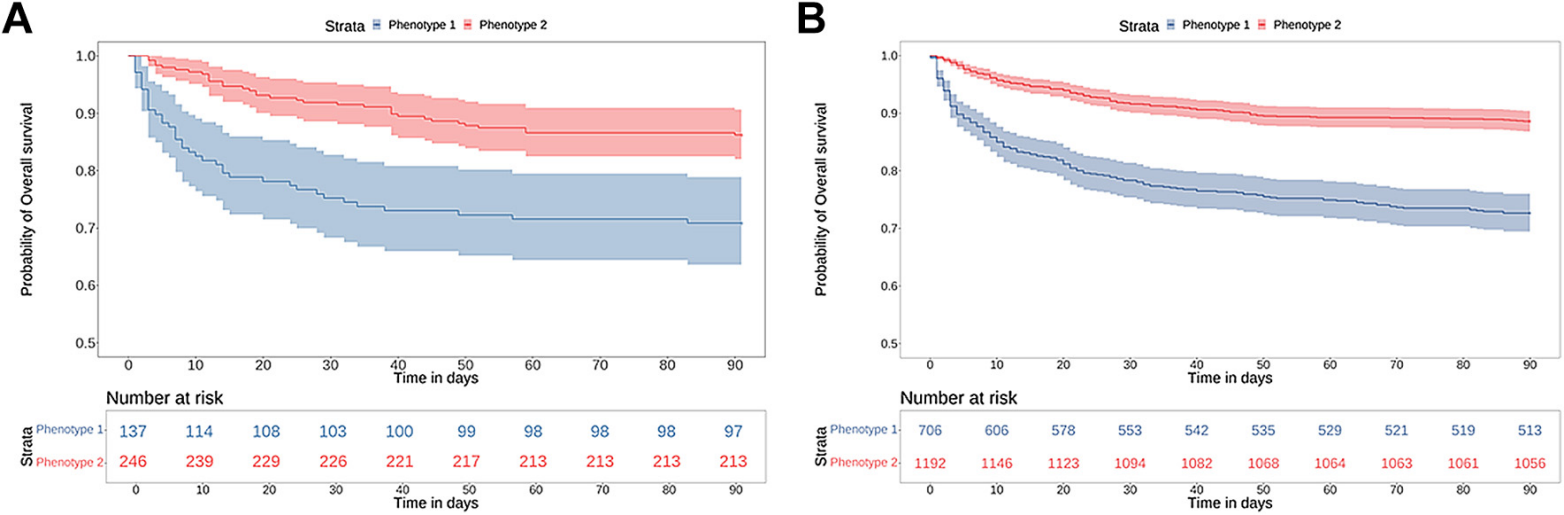
**Survival curves for patients with phenotype 1 versus phenotype 2 in the European cohort (A) and the U.S. cohort (B) of paediatric cancer patients with sepsis.** Shown are the results of Kaplan–Meier analysis of data regarding survival with confidence interval, which were administratively censored at 90 days. Sepsis phenotypes were identified by latent class analysis.

Clinical outcomes differed significantly between the phenotypes. The 90-day mortality was significantly higher in phenotype 1 compared to phenotype 2, 29% versus 13% in the European cohort and 27% versus 11% in the U.S. cohort (p < 0.001 in both cohorts (Table 1; Fig. 2)). No association was found between hospital and mortality in either cohort. After adjusting for organ dysfunction score, haematological cancer, prior stem cell transplantation and age, phenotype 1 was associated with an adjusted odds ratio (aOR) of death at 90-day of 1.9 (1.04–3.34) in the European and 1.6 (1.2–2.2) in the U.S. cohort compared to phenotype 2. In both cohorts, phenotype 1 was associated with increased vasopressor and CRRT use (Table 1). In addition, patients assigned to phenotype 1 in the U.S. cohort received more mechanical ventilation compared to phenotype 2.

In the sensitivity analysis, the Random Forest classifier predicted that 773 and 1125 patients in the U.S. cohort belonged to phenotypes 1 and 2, respectively, based on the characteristics of those patients in European cohort. A total of 605 patients (78%) in phenotype 1 and 1024 (91%) patients in phenotype 2 overlapped between the Random Forest and LCA-based classification in the U.S. cohort (Kappa agreement = 0.7, p < 0.001). Patients who overlapped had a 90-day mortality of 29% in phenotype 1 and 11% in phenotype 2, whereas those in the discordant group (269 patients) had a mortality 16%, which was statistically different from both overlapping groups (p < 0.001).

## Discussion

Using two large, international multicentre cohorts of paediatric cancer patients admitted to the PICU, we identified two distinct phenotypes in paediatric cancer patients with sepsis using routinely available clinical data from the first 24 h of PICU admission. The two phenotypes were reproducible across the two cohorts and independently associated with significantly different clinical outcomes. Phenotype 1 was characterised by increased heart rate, more hypotension, higher CRP and lactate levels, and hepatic, renal, and coagulation dysfunctions compared to phenotype 2. In addition, phenotype 1 was associated with a poor outcome, which was independent from clinically relevant factors like pSOFA score and factors that are historically considered as important risk factors for worse outcomes (e.g., haematological malignancies, prior HSCT).^11^^,^^12^ The identified phenotypes appear to explain some of the heterogeneity of paediatric cancer patients with sepsis, are prognostically informative, and may help in the identification of therapeutic targets in the future.^23^

We found some differences in the phenotype profiles between the two cohorts, similar to what is found in other phenotyping studies in adult and paediatric ARDS and sepsis patients.^13^^,^^14^^,^^24^, ^25^, ^26^ Estimated PaO_2_/FiO_2_ ratios were different between the phenotypes in the U.S. cohort but not in the European cohort. It is possible that the U.S. cohort had a higher incidence of respiratory dysfunction based on the slightly larger proportion of patients receiving mechanical ventilation, or that at least there is a practice that skews towards more mechanical ventilation use in the U.S. PICUs. In any case, it is notable that the estimated PaO_2_/FiO_2_ ratios were generally low in both cohorts (medians in the 150–250 mmHg range), and that the estimated PaO_2_/FiO_2_ ratios did not appear to be very influential in establishing the two phenotypes via the LCA in either cohort (which was done independently) as shown in Fig. 1. In our study, bicarbonate, albumin, BUN, lactate, INR, bilirubin and ALT are common across the two cohorts as phenotype-defining variables.

Our findings are partially aligned with the phenotypes identified in paediatric sepsis patients by Qin and colleagues, although they included only 31 oncology patients, all of which were leukaemia patients.^14^ The characteristics of phenotype 1 shows similarities to the characteristics of Qin’s PedSep-D phenotype, which was characterised by a high number of organ failures, high mortality rates (33.9%) and need for increased organ support. Phenotype 1 also shows similarities with the hyperinflammatory phenotype described in previous studies using LCA in adult and paediatric patients with ARDS,^24^, ^25^, ^26^ particularly the higher CRP levels, hypotension, worse thrombocytopenia, hypoalbuminemia, and worse respiratory failure with hypoxemia. The similarities between the clinical profiles of the phenotypes compared to previous studies could relate to specific differences in inflammatory response (“endotype”) which underlie the two phenotypes. However, whether this hyperinflammatory response is similar to the response found in non-cancer sepsis patients is questionable. The pathophysiological mechanisms in sepsis are complex and even more complex in septic patients with underlying malignancies. Sepsis and cancer share several pathophysiological features. The immune dysfunctions related to sepsis and cancer appear very similar, including reduced cell numbers and functional alterations in innate and adaptive immune cells.^27^ Prolonged periods of neutropenia and suppressing of cellular and humoral immunity render these patients more susceptible to bacterial, viral, and fungal infections. In addition, there is evidence that the presence of neutropenia can enhance inflammation by the inability to down-regulate the activation from pattern-recognition receptors resulting in a dysregulated host response with insufficient clearance of pathogens.^28^ Moreover, it has been shown that patients with neutropenia can generate a profound pro-inflammatory response represented by higher levels of inflammatory mediators than non-neutropenic patients.^29^ Interestingly, the tumour micro-environment shows similarities with that of sepsis, including, among others, the upregulation of checkpoint molecules and upregulation of regulatory T cells. There is an overlap of the pathobiological mechanisms leading to immunological dysfunction that develops in sepsis but is also present in patients with cancer, suggesting mutual interactions between sepsis and cancer.^30^ These findings may suggest differences in underlying sepsis pathogenesis in cancer patients, which may require targeted therapies particular to this patient population. Elucidating the inflammatory response in septic patients with cancer is necessary to determine which anti-inflammatory treatment, i.e., biologicals and monoclonal antibodies targeting specific inflammatory mediators, may benefit these patients.

Our study has several strengths. The phenotypes identified were evident in two large multicentre cohorts. Both cohorts represent the contemporary pattern of oncology patients admitted to the PICU with sepsis, who may occupy a significant proportion of PICU beds in hospitals with oncology services. The high number of included patients in both cohorts permitted independent replication of the analyses. Other studies have used similar data-driven approaches to define phenotypes in sepsis, however these have generally had limited numbers of oncology patients included in the analyses.^13^^,^^14^ To our knowledge, this is the first study including only cancer patients revealing different characteristics of the phenotypes when compared to the non-cancer sepsis patients. Finally, the consistency of our findings in two international cohorts, with 25 PICUs from across Europe and the U.S., strengthen the generalisability of our findings.

Our study has several limitations. We only used routinely collected clinical data from the electronic health records in this retrospective study. No plasma levels of inflammatory biomarkers were available to determine the pathobiological characteristics that may underlie the phenotypes. This raises the possibility that the identified phenotypes represent primarily severity of illness grouping. However, we show that the higher risk phenotype (phenotype 1) was independently associated with mortality after adjusting for organ dysfunction. In addition, we found significant similarities in the clinical characteristics and outcomes when compared to previous phenotyping studies in adult and paediatric ARDS and sepsis patients.^13^^,^^14^^,^^24^, ^25^, ^26^ These studies all showed higher severity of illness with increased organ dysfunction and vasopressor use in the hyperinflammatory group which was consistently associated with worse outcome. Including higher-resolution data in future studies, such as physiological and biological/multi-omics data, could help elucidate the possible underlying pathobiology driving differences in the phenotypes found in the present study.^31^ This may ultimately inform which patients will benefit from or be harmed by specific treatments. Additionally, we only included clinical data available during the first 24 h of PICU admission for pragmatic reasons, but it is possible that the clinical course prior to PICU admission and treatment-specific information, i.e., stages of disease and therapy, could be informative in defining phenotypes of sepsis in children with cancer. However, other risk factors for outcome, such as time to antibiotics, may then be needed as inputs when considering the patient population at the ward who are at risk of developing severe sepsis or septic shock but not yet requiring admission to the PICU.^32^ Thirdly, inclusion of patients in the two cohorts was conducted in different time periods. Both studies were completed before publication of the 2020 Surviving Sepsis Campaign International Guidelines for the management of septic shock and sepsis-associated organ dysfunction in children.^33^ Since this was the first international guideline on management of sepsis specific for children and given heterogeneity of practices across the world, variation in treatment protocols may have existed across the participating PICUs. Finally, missing data were common for some variables included in the LCA model. We used multiple imputation because LCA requires complete datasets. However, the percentages of missing data among the variables used in the LCA model differed among both cohorts and similar results were still found.

Our findings provide a proof-of-concept that paediatric cancer patients with sepsis can be classified into two different phenotypes with prognostic relevance that are highly reproducible in an international cohort of patients. Elucidating the host immune response in these patients with comprehensive biological data is a logical next step. Ultimately, this could help inform future trials by highlighting which phenotypes are more likely to respond to a given intervention, such as anakinra, JAK inhibitors or monoclonal antibodies such as tocilizumab and emapalumab,^34^ which is a crucial step towards personalised medicine. One of the important aspects of this type of preliminary work, is to ensure that oncology patient are both included in future sepsis phenotype-driven clinical trials and -if justified biologically-studied as a subgroup.

## Contributors

RMWvA and LNSP equally conceptualised the study, determined methodology, supervised all aspects of the investigation, curated and analysed the data, validated the data, and completed the original draft. HMlRT contributed to the design and the methodology of the study, and participated in the statistical analysis. RBEvA and LDJB contributed to the methodology, provided statistical support, supervised the statistical analysis, and participated in writing the manuscript. WJET, IJ, CDS, GB, JP, RC, RIC, AMM, AM, MSM, JW, LJS were involved in collecting the data with the help of the study collaborators for the European cohort. CMR, TDB, YL, MRA, EVSF, AG, SLW, LNSP contributed to data extraction for the U.S. cohort. RMWvA, HMlRT, and LNSP verified the data and had access to the raw data. RMWvA, LJS and LNSP developed the first draft of the manuscript. All authors critically revised the manuscript and approved the final manuscript. RMWvA and LNSP had final responsibility for the decision to submit for publication.

## Data sharing statement

Deidentified participant data with a data dictionary can be shared after approval of a proposal with a signed data access agreement and in collaboration with the study group.

## Declaration of interests

LDJB is supported by the Amsterdam UMC fellowship, Health Holland, ZonMW, Innovative Medicine Initiative, and by the Dutch Lung Foundation (Longfonds) through the Dirkje Postma Award and personal fees from Scailyte, Sobi NL, Impentri, Novartis, AstraZeneca and CLS Behring, outside the submitted work. AM received personal fees from Dräger, outside the submitted work. CMR reports a research grant from the Indiana University Pediatric Critical Care, outside the submitted work. TDB reports funding from U.S. AHRQ (R18 HS029298), outside the submitted work. EVSF repots funding from US NIH, outside the submitted work. SLW reports grants from the US NIH/NICHD (R01HD102396 and R01HD105939) and NIH/National Institute of General Medical Sciences (R21GM146159), and royalties from Up To Date, outside the submitted work. LNS reports grants from the US NIH (R01HD105939 and R21GM146159), and personal fees from Celldom, Saccharo, Allyx, outside the submitted work.
