# Supplementary material for: Distinct clinical phenotypes in paediatric cancer patients with sepsis are associated with different outcomes—an international multicentre retrospective study
Source: eClinicalMedicine. 2023 Oct 5;65:102252. doi: 10.1016/j.eclinm.2023.102252 (PMC10570699; doi:10.1016/j.eclinm.2023.102252)
Supplement: Supplementary Appendix, Fig. S1, and Tables S1–S3 [file mmc1.docx]

**Supplementary appendix**

Supplement to: RM Wösten-van Asperen, HM la Roi-Teeuw, RBE van Amstel, et al. Distinct clinical phenotypes in paediatric cancer patients with sepsis are associated with different outcomes – an international multicentre retrospective study.

**Contents**

[**Investigators** 3](#_Toc145578481)

[**Methods** 5](#_Toc145578482)

[**Approach to missing data** 5](#_Toc145578483)

[**Latent class analysis** 5](#_Toc145578484)

[**Table S1.** List of class-defining variables used in the latent class analysis and the proportion of variables missing. 6](#_Toc145578485)

[**Table S2.** Comparison of baseline characteristics of patients between the European and U.S. cohorts. 7](#_Toc145578486)

[**Table S3.** Fit statistics for latent class models from one to five classes in both cohorts 8](#_Toc145578487)

[**Figure S1.** Elbow plot for evaluating the Akaike’s information criteria (AIC) and Bayesian information criteria (BIC) to determine the optimal number of classes in the (A) European and (B) U.S. cohort. 9](#_Toc145578488)

[**Figure S2.** Probabilities of class assignment. Figures show probability of belonging to the class (A for phenotype 1, B for phenotype 2) to which the subject was assigned, by decile of probability. 10](#_Toc145578489)

[**References** 11](#_Toc145578490)

# **Investigators**

**POKER consortium**

Roelie M Wösten-van Asperen,^1^ Hannah M la Roi-Teeuw,^1^ Rombout BE van Amstel,^2^ Lieuwe DJ Bos,^2^ Wim JE Tissing,^3,4^ Iolanda Jordan,^5,6^ Marina Caballero,^5^ Adriana Margarit,^5^ Roi Campos,^5^ Christian Dohna-Schwake,^7,8^ Paula Möller,^7^ Gabriella Bottari,^9^ Carmela Serpe,^9^ John Pappachan,^10^ Roman Crazzolara,^11^ Rosanna I Comoretto,^12^ Angela Amigoni,^12^ Agniezka Mizia-Malarz,^13^ Maria Damps,^13^ Andrea Moscatelli,^14^ Alessia Montaguti,^14^ Giacomo Tardini^14^ María Sánchez-Martín,^15^ Jef Willems,^16^ Luregn J Schlapbach,^17,18^.

**Novel Data-Driven Sepsis Phenotypes in Children study group**

Colin M Rogerson,^19^ Tellen D Bennett,^20^ Yuan Luo,^21^ Mihir R Atreya,^22^ E Vincent S Faustino,^23^ Alon Geva,^24,25^ Scott L Weiss,^26^ Juliane Bubeck-Wardenburg,^27^ Reid Farris,^28^ Mark Hall,^29^ Grace Chong,^30^ Sareen Shah,^31^ Robinder Khemani,^32^ Emily Stroup,^33^ L Nelson Sanchez-Pinto^34^.

^1^Department of Paediatric Intensive Care, University Medical Centre Utrecht/Wilhelmina Children’s Hospital, Utrecht, the Netherlands

^2^Intensive Care, Amsterdam UMC—location AMC, University of Amsterdam, Amsterdam, the Netherlands

^3^Princess Máxima Centre for Pediatric Oncology, Utrecht, the Netherlands

^4^Department of Paediatric Oncology, University of Groningen, University Medical Centre Groningen, Groningen, the Netherlands

^5^Department of Paediatric Intensive Care and Institut de Recerca, Hospital Sant Joan de Déu, University of Barcelona, Barcelona, Spain

^6^Consorcio de Investigación Biomédica en Red de Epidemiología y Salud Pública, Madrid, Spain

^7^Department of Paediatrics I, Paediatric Intensive Care, Children’s Hospital Essen, Germany

^8^West German Centre for Infectious Diseases, University Hospital Essen, University Duisburg-Essen, Essen Germany

^9^Paediatric Intensive Care Unit, Children’s Hospital Bambino Gesù, IRCSS, Rome, Italy

^10^Department of Paediatric Intensive Care, Southampton Children’s Hospital, UK

^11^Department of Paediatrics, Paediatric Intensive Care Unit, Medical University of Innsbruck, Innsbruck, Austria

^12^Department of Paediatric Intensive Care, Department of Woman's and Child's Health, Padua University Hospital, Padua, Italy

^13^Department of Paediatric Oncology, Haematology and Chemotherapy Unit, Medical University of Silesia, Katowice, Poland

^14^Neonatal and Paediatric Intensive Care Unit, IRCCS Istituto Giannina Gaslini, Genova, Italy

^15^Department of Paediatric Intensive Care, Hospital Universitario La Paz, Madrid, Spain

^16^Department of Paediatric Intensive Care, Ghent University Hospital, Ghent, Belgium

^17^Department of Intensive Care and Neonatology and Children’s Research Centre, University Children’s Hospital Zurich, University of Zurich, Zurich, Switzerland

^18^Child Health Research Centre, the University of Queensland, Brisbane, Queensland, Australia

^19^Department of Paediatrics, Division of Critical Care, Indianapolis University School of Medicine, Indianapolis, IN, USA

^20^Departments of Biomedical Informatics and Paediatrics, University of Colorado School of Medicine, Aurora, CO, USA

^21^Department of Preventive Medicine, Northwestern University Feinberg School of Medicine, Chicago, IL, USA

^22^Department of Paediatrics (Critical Care), University of Cincinnati College of Medicine, Cincinnati Children’s Hospital Medical Centre, Cincinnati, OH, USA

^23^Department of Paediatrics, Yale School of Medicine, New Haven, CT, USA

^24^Department of Anaesthesiology, Critical Care, and Pain Medicine and Computational Health Informatics Program Boston Children's Hospital, USA

^25^Department of Anaesthesia, Harvard Medical School, Boston, MA, USA

^26^Division of Critical Care, Department of Paediatrics, Nemours Children’s Health, Delaware, USA

^27^Department of Paediatrics, Washington University School of Medicine, St. Louis, MO, USA

^28^Department of Paediatrics, University of Washington and Seattle Children's Hospital, Seattle, WA, USA

^29^Department of Paediatrics, The Ohio State University and Nationwide Children's Hospital, Columbus, OH, USA

^30^Department of Paediatrics, University of Chicago Pritzker School of Medicine, Chicago, IL, USA

^31^Department of Paediatrics, Cohen Children's Medical Centre, Donald and Barbara Zucker School of Medicine at Hofstra/Northwell, New Hyde Park, NY, USA

^32^Department of Anaesthesiology and Critical Care Medicine, Children's Hospital of Los Angeles, Los Angeles, CA, USA

^33^Department of Pharmacology, Northwestern University Feinberg School of Medicine, Chicago, IL, USA

^34^Department of Paediatrics (Critical Care) and Preventive Medicine (Health & Biomedical Informatics), Northwestern University Feinberg School of Medicine and Ann & Robert H Lurie Children’s Hospital of Chicago, Chicago, IL, USA

# **Methods**

## **Approach to missing data**

Missing data was present in both studies (Table S2). As Latent Class Analysis (LCA) requires complete datasets, we imputed missing data. We assumed that missing data was conditional on observed covariates, and was “missing at random”. We used multiple imputation by chained equations (R-package ‘mice’) which generated values for all missing data using the observed data for all patients.^1^ Imputation was limited to the variables used in the LCA. Five imputation models with a maximum of 50 iterations each were created. The quality of the imputation models was visually assessed using stripplots and density plots for each imputed variable. We presented the results from the first dataset in the manuscript. In addition, the other datasets were evaluated for consistency, according to the approach described by Bos and colleagues.^2^

## **Latent class analysis**

Prior to LCA, we evaluated the correlation between the variables using Spearman’s rank-order statistics. We included only one variable in the LCA for highly correlated variables (correlation coefficient > 0.5). Some variables are age-dependent, including heart rate, blood pressure, respiratory rate, and plasma creatinine. These variables were standardised by transforming the variables to z-scores based on age categories defined by the PODIUM criteria.^3^

The demographic, clinical and laboratory variables included as potential class defining variables in the latent class analysis (LCA) were guided by the studies in adults. We used log or square root transformation for continuous variables that were skewed. As scales of each measure varied considerably, all continuous variables were standardized by creating Z scores before LCA analysis, as done previously.^4,5^

LCA was performed with the FLXMCmvcombi model of the flexmix package in R.^6^

**Random forest classifier**

A Random Forest classifier was trained in the European cohort with the outcome being the phenotype and the features being the same class-defining variables used in the LCA. We then used the resulting Random Forest model to assign a predicted phenotype in the U.S. cohort and assessed the inter-rater agreement between the predicted phenotype and the LCA-based phenotype. The Random Forest was trained with the Caret package using standard parameters, including 500 trees, using 10-fold cross-validation for training control, using the receiving operating characteristic (ROC) curve as the optimization metric, and using the square root of the model variables count as the number of variables randomly sampled at each split time.

# **Table S1.** List of class-defining variables used in the latent class analysis and the proportion of variables missing.

| **Variables** | **Number Missing (%)** | |
| --- | --- | --- |
|  | **European cohort (n = 383)** | **US cohort (n = 1898)** |
| Age | 0 | 0 |
| Albumin | 158 (41·3) | 393 (20·7) |
| ALT | 69 (18·0) | 732 (38·6) |
| Bicarbonate | 101 (26·4) | 82 (4·3) |
| Bilirubin | 109 (28·5) | 741 (39) |
| BUN | 71 (18·5) | 77 (4·1) |
| C-reactive protein | 125 (32·6) | 1428 (75·2) |
| Creatinine | 51 (13·3) | 83 (4·4) |
| GGT | 101 (26·4) | 1707 (89·9) |
| Glucose | 45 (11·7) | 140 (7·4) |
| Haemoglobin | 40 (10·4) | 78 (4·1) |
| Haematological cancer | 0 | 0 |
| Heart rate | 12 (3·1) | 0 |
| INR | 181 (47·3) | 783 (41·3) |
| Lactate | 62 (16·2) | 691 (36·4) |
| Leucocyte count | 89 (23·2) | 103 (5·4) |
| PCO_2_ | 99 (25·8) | 538 (28·3) |
| PaO_2_/FiO_2_ ratio* | 140 (36·6) | 511 (26·9) |
| Platelet count | 76 (19·8) | 98 (5·2) |
| Potassium | 50 (13·1) | 60 (3·2) |
| Prior HSCT | 0 | 0 |
| Respiratory rate | 18 (4·7) | 0 |
| Sex | 0 | 0 |
| Sodium | 40 (10·4) | 15 (0·8) |
| Systolic blood pressure | 13 (3·4) | 0 |
| Temperature | 20 (5·2) | 99 (5·2) |

Abbreviations: ALT = Alanine transaminase. BUN = Blood urea nitrogen. GGT = Gamma-glutamyltransferase. HSCT = Haematopoietic Stem Cell Transplantation. ^*^If only oxygen saturation was available, it was converted to PF, as described previously by Khemani et al^7^.

# **Table S2.** Comparison of baseline characteristics of patients between the European and U.S. cohorts.

|  | **European cohort**  **(n=383)** | **U.S. cohort**  **(n=1898)** |
| --- | --- | --- |
| Age in years, median (IQR) | 8·6 (3·7–13·9) | 8·0 (3·5–13·6) |
| Sex, male, No. (%) | 217 (56·7) | 1,063 (56·0) |
| Oncological diagnosis, No. (%)  Haemato–oncological  Solid tumour  Brain & Central nervous system tumour  Other | 281 (73·4)  48 (12·5)  51 (13·3)  3 (0·8) | 1,154 (60·8)  356 (18·8)  332 (17·5)  41 (2·2) |
| Prior HSCT, No. (%) | 112 (29·2) | 320 (16·9) |
| PELOD–2 score, median (IQR) | 5 (2–7) | 7 (5–10) |
| pSOFA score, median (IQR) | 9 (6–11) | 8 (6–10) |
| Highest heart rate (bpm) | 152 (130–171) | 156 (137–177) |
| Lowest systolic pressure (mmHg) | 85 (73–98) | 81 (69–91) |
| Highest respiratory rate (bpm) | 38 (29–50) | 41 (32–54) |
| Highest temperature (Deg. C) | 38·0 (37·2–38·8) | 38·8 (37·9–39·9) |
| Lowest estimated PaO_2_/FiO_2_ | 232·5 (140·7–442·9) | 151·0 (93·0–232·5) |
| Highest PCO_2_ (mmHg) | 45·9 (40·2–51·0) | 45·0 (39·0–54·0) |
| Highest glucose (mmol/L) | 7·7 (6·5–10·3) | 8·5 (6·6–12·0) |
| Highest potassium (mmol/L) | 4·2 (3·8–4·7) | 4·0 (3·5–4·5) |
| Highest sodium (mmol/L) | 136 (133–139) | 135 (132–138) |
| Highest creatinine (µmol/L) | 39·0 (26·0–65·6) | 43·3 (26·5–70·7) |
| Highest BUN (mmol/L) | 5·9 (3·5–9·5) | 4·6 (2·9–8.2) |
| Highest albumin (g/L) | 27·0 (20·8–31·4) | 27·0 (23·0–32·0) |
| Lowest bicarbonate (mmol/L) | 21·9 (18·9–25·2) | 21·0 (17·0–24·0) |
| Highest lactate (mmol/L) | 1·8 (1·1–2·9) | 1·9 (1·1–3·9) |
| Highest bilirubin (µmol/L) | 17·0 (8·9–36·0) | 20·5 (8·6–48·6) |
| Highest ALT (U/L) | 41·5 (21·0–82·8) | 47·0 (25·0–119·8) |
| Highest GGT (U/L) | 79·0 (35·0–207·0) | 76·0 (40·5–175·0) |
| Highest INR | 1·3 (1·1–1·6) | 1·4 (1·2–1·8) |
| Lowest WBC (10^9^/L) | 1·6 (0·3–9·0) | 3·1 (0·3–9·2) |
| Lowest platelets (10^9^/L) | 25·0 (11·0–65·5) | 48·0 (24·0–94·0) |
| Lowest haemoglobin (mmol/L) | 5·0 (4·4–5·7) | 5·3 (4·5–6·1) |
| Highest CRP (mg/L) | 159·5 (58·5–254·8) | 81·0 (32·0–211·0) |
| Abbreviations: ALT = Alanine transaminase. BUN = Blood urea nitrogen. CRP = C-reactive protein. GGT = Gamma-glutamyltransferase. HSCT = Haematopoietic Stem Cell Transplantation. IQR = interquartile range. PELOD = Paediatric Logistic Organ Dysfunction. pSOFA = Paediatric Sequential Organ Failure Assessment. VIS= Vaso-active inotropic score. WBC = White blood cell. | | |

# **Table S3.** Fit statistics for latent class models from one to five classes in both cohorts

| **European cohort** | **Bayesian Information**  **Criterion** | **Akaike’s Information Criterion** | **Entropy** | **Number of individuals per class or phenotype** | | | | |
| --- | --- | --- | --- | --- | --- | --- | --- | --- |
|  | | | | 1 | 2 | 3 | 4 | 5 |
| 1 class | 26709·99 | 26516·53 |  | 383 |  |  |  |  |
| 2 classes | 25793·44 | 25402·58 | 0·875 | 137 | 246 |  |  |  |
| 3 classes | 25535·17 | 24946·91 | 0·902 | 95 | 171 | 117 |  |  |
| 4 classes | 25411·35 | 24625·69 | 0·904 | 79 | 74 | 93 | 137 |  |
| 5 classes | 25387·16 | 24404·10 | 0·915 | 57 | 160 | 53 | 66 | 47 |

| **U.S. cohort** | **Bayesian Information**  **Criterion** | **Akaike’s Information Criterion** | **Entropy** | **Number of individuals per class or phenotype** | | | | |
| --- | --- | --- | --- | --- | --- | --- | --- | --- |
|  | | | | 1 | 2 | 3 | 4 | 5 |
| 1 class | 311498·3 | 311237·5 |  | 1898 |  |  |  |  |
| 2 classes | 184714·5 | 184187·4 | 0·999 | 706 | 1192 |  |  |  |
| 3 classes | 184393·9 | 183866·8 | 0·999 | 707 | 234 | 957 |  |  |
| 4 classes | 180196·5 | 179269·9 | 0·993 | 164 | 117 | 106 | 1511 |  |
| 5 classes | 183219·8 | 182559·5 | 0·994 | 114 | 94 | 85 | 182 | 1423 |


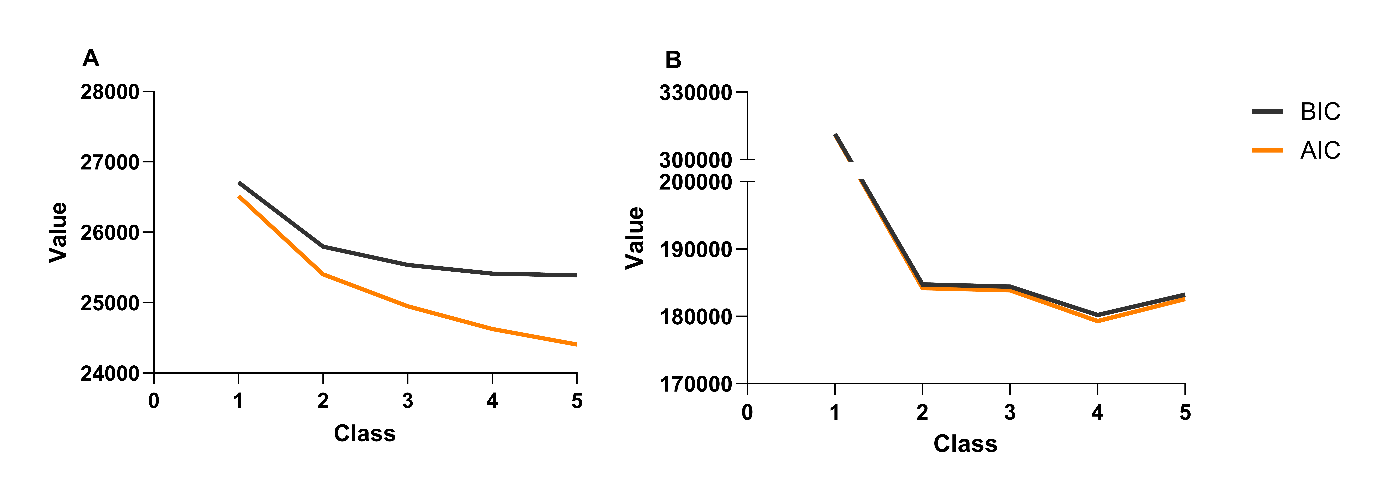


# **Figure S1.** Elbow plot for evaluating the Akaike’s information criteria (AIC) and Bayesian information criteria (BIC) to determine the optimal number of classes in the (A) European and (B) U.S. cohort.

# **Figure S2.** Probabilities of class assignment. Figures show probability of belonging to the class (A for phenotype 1, B for phenotype 2) to which the subject was assigned, by decile of probability.
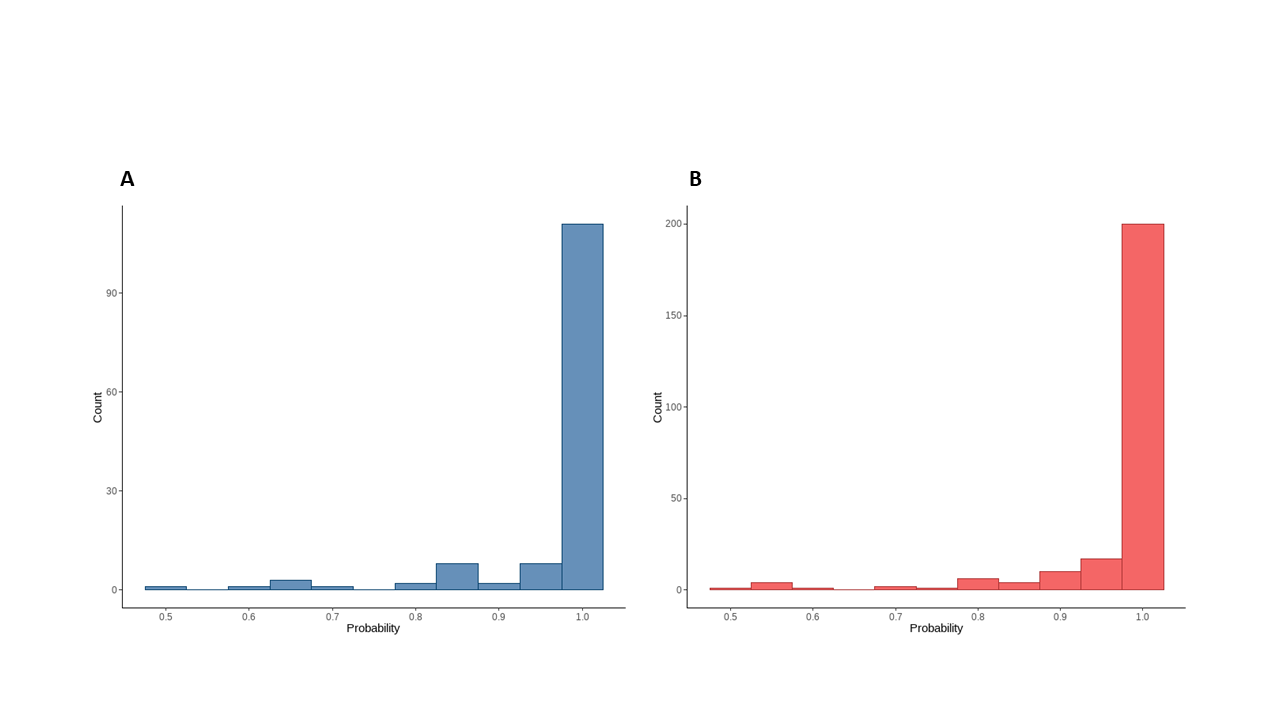


# **References**

1. van Buuren S, Boshuizen HC, Knook DL. Multiple imputation of missing blood pressure covariates in survival analysis. *Stat Med* 1999; **18**: 681-94.
2. Bos LDJ, Sjoding M, Sinha P, et al. Longitudinal respiratory subphenotypes in patients with COVID-19-related acute respiratory distress syndrome: results from three observational cohorts *Lancet Respir Med* 2021; **9**: 1377-86.
3. Bembea MM, Agus M, Akcan-Arikan A, et al. Pediatric Organ Dysfunction Information Update Mandate (PODIUM) Contemporary Organ Dysfunction Criteria: Executive Summary. *Pediatrics* 2022; **149**: S1-S12.
4. Marshall A, Altman DG, Holder RL. Comparison of imputation methods for handling missing covariate data when fitting a Cox proportional hazards model: a resampling study. *BMC Med Res Methodol* 2010; **10**: 112.
5. Marshall A, Altman DG, Royston P, Holder RL. Comparison of techniques for handling missing covariate data within prognostic modelling studies: a simulation study. *BMC Med Res Methodol* 2010; **10**: 7.
6. Leisch F. FlexMix: A general framework for finite mixture models and latent class regression in R. *Journal of Statistical Software* 2004; **11**: 1-18.
7. Khemani RG, Patel NR, Bart RD 3rd, Newth CJL. Comparison of the pulse oximetric saturation/fraction of inspired oxygen ratio and the PaO2/fraction of inspired oxygen ratio in children. *Chest* 2009; **135**: 662-68.
